# Photoelectrochemical Green Hydrogen Production Utilizing ZnO Nanostructured Photoelectrodes

**DOI:** 10.3390/mi14051047

**Published:** 2023-05-14

**Authors:** Sameerah I. Al-Saeedi

**Affiliations:** Department of Chemistry, College of Science, Princess Nourah Bint Abdulrahman University, P.O. Box 84428, Riyadh 11671, Saudi Arabia; sialsaeedi@pnu.edu.sa

**Keywords:** ZnO photoelectrodes, nanorods, nanoparticulate, photoelectrochemical hydrogen production, conversion efficiencies, stability

## Abstract

One of the emerging and environmentally friendly technologies is the photoelectrochemical generation of green hydrogen; however, the cheap cost of production and the need for customizing photoelectrode properties are thought to be the main obstacles to the widespread adoption of this technology. The primary players in hydrogen production by photoelectrochemical (PEC) water splitting, which is becoming more common on a worldwide basis, are solar renewable energy and widely available metal oxide based PEC electrodes. This study attempts to prepare nanoparticulate and nanorod-arrayed films to better understand how nanomorphology can impact structural, optical, and PEC hydrogen production efficiency, as well as electrode stability. Chemical bath deposition (CBD) and spray pyrolysis are used to create ZnO nanostructured photoelectrodes. Various characterization methods are used to investigate morphologies, structures, elemental analysis, and optical characteristics. The crystallite size of the wurtzite hexagonal nanorod arrayed film was 100.8 nm for the (002) orientation, while the crystallite size of nanoparticulate ZnO was 42.1 nm for the favored (101) orientation. The lowest dislocation values for (101) nanoparticulate orientation and (002) nanorod orientation are 5.6 × 10^−4^ and 1.0 × 10^−4^ dislocation/nm^2^, respectively. By changing the surface morphology from nanoparticulate to hexagonal nanorod arrangement, the band gap is decreased to 2.99 eV. Under white and monochromatic light irradiation, the PEC generation of H_2_ is investigated using the proposed photoelectrodes. The solar-to-hydrogen conversion rate of ZnO nanorod-arrayed electrodes was 3.72% and 3.12%, respectively, under 390 and 405 nm monochromatic light, which is higher than previously reported values for other ZnO nanostructures. The output H_2_ generation rates for white light and 390 nm monochromatic illuminations were 28.43 and 26.11 mmol.h^−1^cm^−2^, respectively. The nanorod-arrayed photoelectrode retains 96.6% of its original photocurrent after 10 reusability cycles, compared to 87.4% for the nanoparticulate ZnO photoelectrode. The computation of conversion efficiencies, H_2_ output rates, Tafel slope, and corrosion current, as well as the application of low-cost design methods for the photoelectrodes, show how the nanorod-arrayed morphology offers low-cost, high-quality PEC performance and durability.

## 1. Introduction

Photoelectrochemical (PEC) catalytic hydrogen generation has gained global recognition as one of the most hopeful methods for utilizing the world’s biggest sustainable solar energy source [1]. Since Fujishima and Honda used TiO_2_ photoelectrodes for PEC water splitting, numerous semiconductors have been used for direct solar energy gathering for the production of green hydrogen via PEC water splitting [2,3,4]. PEC green hydrogen production has several benefits, including an appropriate hydrogen production rate, high-purity hydrogen production, fast system reaction, and high safety [5]. Photoelectrodes based on metal oxides can be readily synthesized with appropriate morphology, resulting in increased surface area for enhanced green hydrogen generation [6].

Nanostructures have sparked the attention of scientists and engineers worldwide due to their unique chemical, optical, and electronic properties, which lend themselves to a broad range of novel applications. Zinc oxide (ZnO) nanostructures are widely used in many applications due to their broadband gap energy (3.1–3.4 eV), high exciton binding energy (60 meV), and excellent thermal and chemical stability [7,8,9]. Furthermore, ZnO is non-toxic and bio-safe, with no environmental or health hazards associated with large-scale production. ZnO is also available in a variety of nanostructured forms [10,11]. ZnO nanoparticles are currently used in a wide range of applications, including field emission, gas sensors, solar cells, field-effect transistors, antimicrobial activities, self-cleaning, light-emitting diodes, laser systems, and photocatalysts [12,13,14,15,16,17,18]. The photocatalytic effectiveness of ZnO surfaces in the breakdown of dangerous dye molecules in both basic and acidic media has been enhanced [19]. ZnO surfaces have a thermal diffusivity of (4.35–5.03) × 10^−2^ cm^2^/s and an electrical impedance ranging from 10^−4^ to 10^12^ Ω·cm [20]. The spontaneous exciton binding energy of ZnO in superlattices can be increased from 60 to >100 meV at room temperature [21]. The ZnO semiconductor material’s distinctive physicochemical and optoelectronic properties continue to stimulate the interest of many industrial and technological applications, including photoelectrochemical water splitting (PEC-WS) [22]. Because of their capacity to block UV radiation, ZnO nanoparticles (NPs) have been used in cosmetics and coatings [23,24]. The construction of nanorods and nanowires, furthermore, can expand the absorption zone to extended wavelengths [22,25,26]. Grushevskaya et al. claim that ZnO microrods and nanorods with high crystallinity are capable of transferring electrons across lengthy distances at an acceptable rate without encountering recombination with holes [27]. The capacity of ZnO to photodegrade the organic contaminants from water is improved when these electrons are absorbed by the dissolved oxygen on its surface, producing hydrogen peroxide and a hydroxyl radical. Additionally, Almamari et al. reported that ZnO nanostructure optimization can improve the optical absorbance and photochemical performance of ZnO [28]. As a result, it is critical to understand how turning ZnO nanomorphology with good crystallinity from nanoparticulates to nanorods affects the PEC performance and durability of the developed photoelectrodes. The PEC-WS focuses on the regulated development of ZnO nanostructures. Different techniques can be used to create various nanomorphologies of ZnO. Magnetron sputtering, atomic layer deposition, heat vaporization, vapor phase epitaxy, solid-state reactivity, and hydrothermal techniques are among them [29,30,31,32,33]. These techniques, however, necessitate ultra-high vacuum, costly apparatuses, high operating temps, catalysts, or the use of noxious gas compounds. However, there is a high demand for low-cost, mass-produced ZnO nanostructured surfaces for industrial applications of PEC-WS, particularly ZnO nanorod array based coatings. In comparison to the methods mentioned above, preparing ZnO nanostructure films using chemical bath deposition (CBD) and spray pyrolysis is more appealing, due to their low cost, simplicity, rapidity, ability to control the morphology and orientation of ZnO by tuning process parameters, working at low temperatures (<100 °C), and applicability for large-scale production using the surfaces of a variety of substrates [34,35,36].

Previous studies concentrated on the use of CBD to regulate the shape of ZnO nanostructures by varying the growing period, concentration, and temperature of the solution [37,38,39]. Furthermore, Shaban et al. reported the production of ZnO thin films using spray pyrolysis with flow rates ranging from 20 to 100 L/min, and the films were used for self-cleaning [36]. These nanoparticles were not used in the creation of PEC hydrogen. ZnO nanostructured photoelectrodes are developed in this work using CBD and spray pyrolysis under previously optimized circumstances [36,39]. Various characterization methods are used to investigate morphologies, structures, elemental analysis, and optical characteristics. Under white light and monochromatic light irradiation, the photoelectrochemical generation of H_2_ using the proposed photoelectrodes is investigated. The conversion efficiencies and quantity of hydrogen moles are determined using the PEC process’ J_ph_-V and J_ph_-t curves. The durability and reusability of the used electrodes are also assessed and linked with the morphologies, structures, and optical characteristics of the developed electrodes.

## 2. Experimental Details

### 2.1. Materials

Zinc acetate dihydrate (Zn(CH_3_COOH).2H_2_O, 99%), zinc sulfate (ZnSO_4_, 99%), ammonia (NH_4_OH), sulfuric acid (H_2_SO_4_, 95%), sodium hydroxide (NaOH, 99.6%), hexamethylene tetraamine (C_6_H_12_N_4_ (HMT), ≥99%), hydrochloric acid (HCl, 98%), and sodium sulfite heptahydrate (Na_2_SO_3_.7H_2_O, 99%) were purchased from Alfa Aesar, Haverhill, MA, USA. These chemicals were used exactly as they were delivered by the vendors. Microscopic glass slides (size: 75 mm × 25 mm × 1.0 mm) were used after pre-cleaning as glass substrates for the growth of ZnO nanorod-arrayed film and ZnO nanoparticulate film. The glass substrates were cleaned in boiled dilute sulfuric acid (1:10 *v*/*v*) for 45 min before being completely rinsed in ethanol, acetone, and de-ionized (DI) water for 10 min at 90 °C. Lastly, the glass substrates were dried in a kiln at 70 °C for 20 min.

### 2.2. Deposition of ZnO Nanorod-Arrayed Film

The ZnO nanorod-arrayed film was formed on the cleaned glass substrates using a two-step process that included seed layer creation by dip-coating and subsequent development using chemical bath deposition (CBD).

#### 2.2.1. Deposition of ZnO Seed Layer

At room temperature (20 °C), ZnSO_4_ analytical chemicals were dissolved in reduced ammonia to produce a 0.1 M zinc ammonium complex solution of volume 50 mL, which was then used as a cationic precursor. As an anionic predecessor, a 50 mL double-distilled water (DDW) maintained at 80 °C was used. SILAR growth is a four-step process that begins with submerging a cleaned glass substrate in cationic and anionic solutions and ends with rinsing it in DDW at room temperature. The zinc ammonia adsorbed substrate was then submerged for 20 s in a beaker holding 50 mL of DDW, where the adsorbed zinc ammonia complex was transformed into zinc hydroxide (Zn(OH)_2_). The Zn(OH)_2_ coated substrate was then ultrasonically agitated for 60 s at 20 °C to remove weakly bound zinc hydroxide (Zn(OH)_2_) molecules. Lastly, the zinc hydroxide coated substrate was submerged in a DDW bath at 80 °C for 20 s, where Zn(OH)_2_ was converted to a solid ZnO layer. The as-prepared ZnO seed layer sheets were annealed for 1 h at 300 °C. ZnO seed layer films were produced at 20 cycles in the current study. The choice to use 20 cycles was made based on Abdulrahman et al.’s [40] work, which shows that the seed layer that deposited for 15 cycles and 20 cycles is more crystalline with low dislocation densities and may be used for ZnO nanorods’ growth.

#### 2.2.2. Growth of ZnO Nanorod-Arrayed Film Using CBD

The chemical bath was made by normally combining an equimolar (0.05 M) solution of zinc acetate dihydrate, Zn(CH_3_COOH)·2H_2_O, with hexamethylene tetra ammine (HMT, 0.05 M) dissolved in 20 mL of deionized water (DI) water at 80 °C. There was no precipitation noted when these two solutions were mixed. After 20 min of continuous stirring, white precipitation was noticed. The pH of the process was kept constant at 6.6 using 0.1 M NaOH solution and the deposition was carried out for 2 h. The temperature of the water bath was permitted to drop down to atmospheric temperature after the deposition condition was completed. Lastly, the deposited film was carefully removed from the aqueous solution and washed several times with DI water and acetone to eliminate leftover salts before being dried at room temperature for further analysis.

### 2.3. Deposition of ZnO Nanoparticulate Film Using Spray Pyrolysis

A basic, inexpensive, and homemade spray pyrolysis device was used to deposit ZnO nanoparticulate film on the pre-cleaned glass substrates. A homogenous and stable precursor solution was created by soaking 0.01 M zinc acetate dihydrate in 50 mL DI water at 20 °C for 20 min. According to Lee et al.’s work [41], 0.01 M of ZnO was selected to make ZnO films with nanoparticulate surfaces without agglomerations or clusters. This solution was sprayed for 15 min at 300 °C through a 0.2 mm nozzle onto a heated pre-cleaned glass substrate. The nozzle–substrate distance was set to 50 cm, with pressurized air serving as the carrier gas. The solution flow rate was kept at 80 µL/min.

### 2.4. Sample Characterization

Morphological examinations of the manufactured nanostructured films were performed using field emission–scanning electron microscopy (FE-SEM, model: ZEISS SUPRA 55 VP and ZEISS LEO, Gemini Column). The elemental composition was investigated using energy-dispersive X-ray technology (EDX; Oxford Link ISIS 300 EDX) up to 10 keV. For crystallographic property determination of the prepared ZnO films, high-resolution X-ray diffraction (XRD, Philips X’PertPro MRD) was used with Cu K radiation (λ = 1.5418 Å) in a scan range of 10° to 70° with a scan rate of 0.05°/min. At RT, optical absorbance spectra in the 200–1100 nm region were recorded using a UV/VIS/NIR Perkin Elmer 950 double-beam spectrometer.

### 2.5. PEC Measurements

At ambient temperature (25 °C), photoelectrochemical H_2_ production observations were performed in 50 mL of 0.5 M Na_2_SO_3_.7H_2_O solution with a sweep rate of 1 mV/s. In the water-splitting test response, a two-electrode cell was used, with the developed photoelectrode having a 1 cm^2^ surface area and the counter electrode being a Pt electrode. A 400 W mercury–xenon lamp (Newport, 66142-500HX-R07, Newport, UK) was used to provide standard white light illumination of 100 mW cm^−2^ on the electrode surface. The impacts of incident wavelength (390–636 nm) and temporal stability on water splitting were investigated. The PEC current density–voltage (J_ph_-V) curves were measured using voltage linear sweep mode with a sweep rate of 10 mV/s from 0 to 1 V. The current density–time (I-t) curves were measured using the amperometry mode at 1 V for 900 s.

## 3. Results and Discussion

### 3.1. Sample Characterization

#### 3.1.1. Surface Morphology and Elemental Composition

**Figure 1:**
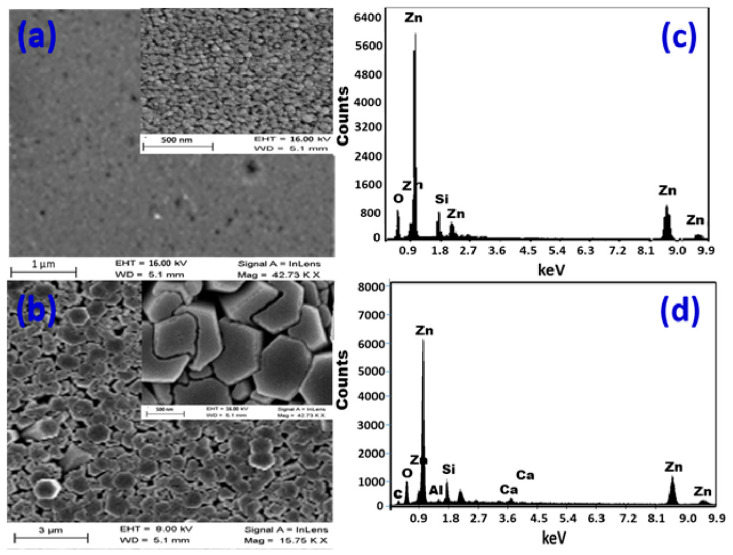
SEM images and EDX elemental analysis for (**a**,**c**) nanoparticulate film and (**b**,**d**) hexagonal nanorod array.

Figure 1a depicts the morphology of a ZnO layer that was formed at 80 µL/min for 15 min. The glass surface is densely packed with nanoparticles that are almost evenly dispersed. The nanoparticles self-assemble to create nanopores in some locations. The pores that develop have sizes of less than 28 nm. Figure S1 (Supplementary Materials) shows the pore diameter distribution, where the pore diameter ranged from 10 nm to 28 nm with an average value of 19 nm. The estimated pore density is (30.9 ± 1.1) × 10^9^ pore/cm^2^. The formed layer has a thickness of ~300 nm. Figure 1b shows SEM images of the CBD-deposited ZnO thin film with a high density and aspect ratio of hexagonal nanorods vertically oriented on the glass substrate. The rods are shaped into segments of varying diameters. The hexagonal nanorods have an average diameter of ~700 nm. The simultaneous development of this high density of nanorods causes compression microstrains, which destructs some nanorods. As a consequence, some nanorods’ shapes are deformed, and others are broken. The microstrains actually exaggerate the atomic disorder caused by a structural defect found at the surface of the nanocrystallite because of the high atomic surface/volume ratio of nanorods. As the crystallites become smaller, the microstrain becomes bigger and the atoms are pulled away from their ideal positions by the microstrain because of structural imperfections such as stacking faults, twins, grain boundaries, and/or dislocations. Higher surface area may result from the splitting and destruction of nanorods, which might enhance ZnO nanorods’ catalytic efficacy.

The samples were examined using EDX to determine the elemental makeup of the manufactured ZnO nanostructures, as shown in Figure 1c,d. Figure 1c shows the presence of Zn and O signals in the EDX spectrum of a nanoparticulate ZnO film, which correlates to the typical makeup of ZnO. According to the precise EDX study, there is 84.7% Zn and 10% O. Because the EDX interaction volume (≥1 μm) is larger than the film thickness, the Si (5.3%) signal in the EDX chart was identified from the glass substrate.

**Figure 2:**
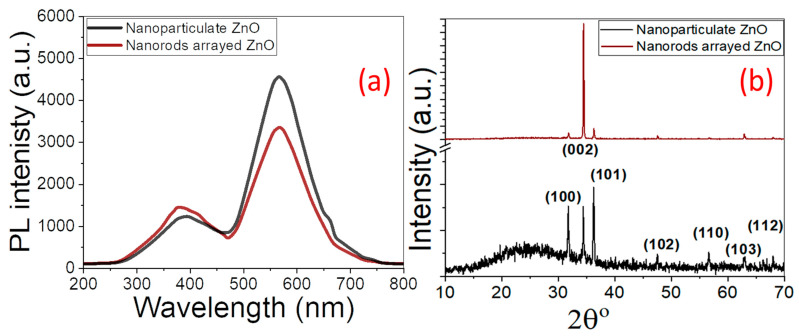
(**a**) PL spectra and (**b**) XRD patterns of nanoparticulate and nanorod-arrayed ZnO films.

Figure 1d shows the EDX spectrum of a nanorod-arrayed film, which clearly shows the existence of Zn signals at 1.0, 8.6, and 9.6 keV, as well as an O signal at 0.525 eV. With 84.6% Zn and 15.4% O, the oxygen percentage in ZnO is lower than the stoichiometric ratio of O atoms, but it still shows good purity, according to quantitative analysis. Furthermore, Si, Ca, and Al signals from the glass substrate are identified, suggesting that the ZnO layer thickness is less than the EDX interaction volume (≥1 μm). While the Zn% is about the same in both films, the O% is lower in the nanoparticulate film than in the nanorod-arrayed film due to the oxygen vacancies. The reduction of oxygen vacancies may be brought on during the annealing process by the interaction of oxygen and uncoordinated zinc at the nanorods’ surfaces, as well as the migration of oxygen ions into the oxygen-deficient regions of the nanorods’ crystal structure [42]. This also was confirmed by the measured photoluminescence (PL) spectra of the nanorod-arrayed and nanoparticulate ZnO films as shown in Figure 2a. These PL spectra were measured using the 325 nm line of a He-Cd laser as an excitation source at room temperature. ZnO nanorod-arrayed film and ZnO nanoparticulate film exhibited a weak band edge emission at 374 nm resulting from free-exciton annihilation [43] and a very strong and broad yellow-orange emission at ~570 nm attributed to oxygen vacancy [44]. The ZnO nanorod-arrayed film showed higher intensity than that obtained from nanoparticulate film at 374 nm, but lower intensity at 570 nm. This indicates that the nanorod-arrayed film had a lower oxygen vacancy than the nanoparticulate film.

#### 3.1.2. XRD Structural Properties

Figure 2b shows the XRD patterns of ZnO nanoparticulate and nanorod-arrayed ZnO films. The films showed polycrystalline natures, where the diffraction XRD peaks become stronger and sharper for nanorod-arrayed film than nanoparticulate film. The XRD pattern of nanoparticulate ZnO film shows a strong (101) diffraction peak at 2θ = 36.2°, whereas the nanorod-arrayed film pattern shows a strong (002) diffraction peak at 2θ = 34.5°. The observed diffraction peaks for the fabricated ZnO nanostructures are attributed to hexagonal wurtzite ZnO phases with c-axis orientation (space group P63mc). Table 1 shows the positions of all peaks and their associated diffraction orientation planes. Each Zn^2+^ ion in hexagonal wurtzite ZnO is tetrahedrally bound to four O^2−^ ions and vice versa. The development of ZnO nanorods is then preferred to be perpendicular to the material surface. There are no impurity phases identified in the XRD patterns, indicating the production of pure crystalline ZnO films.

As shown in Table 1, the Scherer equation was used to determine the average crystallite size (DC) along each direction [45]. For nanoparticulate ZnO, the crystallite size was 47.5, 75.9, and 42.1 nm for the (100), (002), and (101) orientations, respectively. For the (002) orientation of the nanorod-arrayed film, the crystallite size was 100.8 nm. The minimum dislocation density was estimated (δ = 1/D_C_^2^) and reported in Table 1. The values for (101) of nanoparticulate and for (002) of nanorods were 5.6 × 10^−4^ and 1.0 × 10^−4^ dis/nm^2^, respectively. In general, the density of dislocations for ZnO nanorods is lower than the density of dislocations for the nanoparticulate film. According to C.-H. Cho and H. Cho, dislocation density is the single major component that influences hardness and tensile strength; nevertheless, dislocation density and arrangement both impact electrical conductivity [46]. Only Matthiessen’s rule addresses the loss in electrical conductivity caused by dislocations brought on by macroscale electron scattering. However, Ishida et al. [47] explained how dislocations and their motions affect electric conductance at the nanoscale, and how oscillations in conductance are brought on by recurring and discontinuous dislocation movements. So, the nanorods’ morphology can be more stable and more suitable for PEC application. The texture coefficient (TC) was calculated to determine the favored crystallographic orientations of the ZnO films. Table 1 displays the TC values of the (hkl) planes [48]. If TC_(hkl)_ ≥1, this indicates the preferential growth of the crystallites perpendicular to the (hkl) plane. For the nanoparticulate ZnO film, it can be observed from Table 1 that the TC_(101)_ > TC_(002)_ > TC_(100)_ > 1. For the nanorod-arrayed ZnO film, the TC_(002)_ > 1.83, which implies preferential growth of ZnO crystals along axis c. The greater quantity of nanograins along the (002) plane accounts for the rise in favored orientation, i.e., this demonstrates that the grains during spray pyrolysis of the ZnO nanoparticulate film are aligned with their preferred (101), (002), and (100) planes, in contrast to the majority of the grains of the CBD-deposited nanorods, which are only oriented along the (002) plane. In other words, the method used to grow ZnO nanorods gradually promotes the growth of the (002) plane preferentially and prevents the formation of Zn dendrites, resulting in the formation of flat and compact hexagonal nanorods, which may increase the stability of the electrode during prolonged use in the PEC process.

Table 1 also shows the computed and displayed lattice parameters (a, c), unit cell volume (V), Zn-O bond length, and in-plane stress of the ZnO films [49,50,51,52]. The nanorods have a marginally lower c-parameter (5.198 Å) than the nanoparticulate (5.212 Å). Similarly, the bond length is reduced marginally from 1.683 Å to 1.680 Å. This is complemented by a negative in-plane stress (−4.69 × 10^8^ N/m^2^) for nanoparticulate and a positive value (7.97 × 10^8^ N/m^2^) for nanorods. The crystallites are under compressive stress if the expected stress values are negative, whereas the crystallites are under tensile stress if the estimated stress values are positive.

#### 3.1.3. Optical Properties and Band Gap Calculation

**Figure 3:**
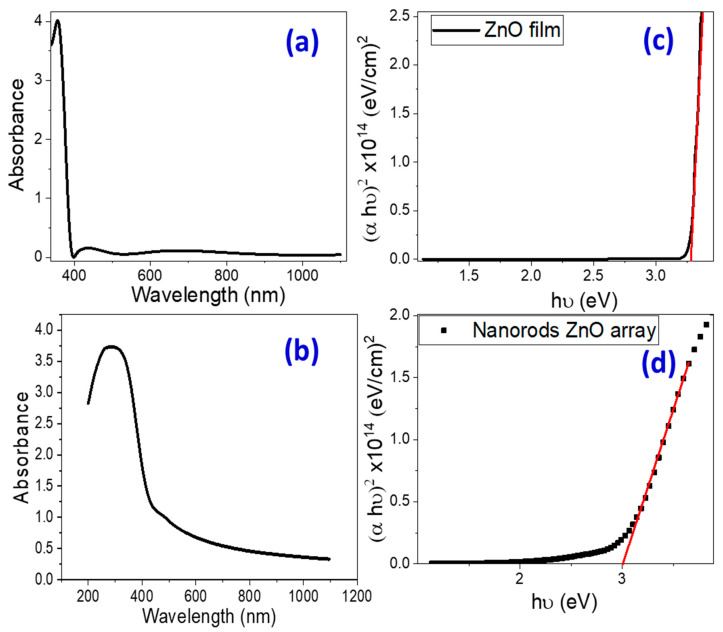
(**a**,**b**) Absorbance spectra and (**c**,**d**) Tauc’s plots for band gap determination for nanoparticulate and nanorod-arrayed ZnO films. Red line represents the linear fitting of the linear segment of Tauc’s plot (Black curve).

UV-Vis absorption spectroscopy is an effective tool for investigating the optical characteristics and band structure of semiconductor nanomaterials. Figure 3a,b depicts the absorbance spectra of pure ZnO nanoparticulate and nanorod-arrayed films deposited onto glass substrates. For nanoparticulate ZnO film, there is a strong and sharp absorption band at 355 nm and a powerful broad absorption band extending from 200 to 450 nm for ZnO nanorod-arrayed film.

By applying the Tauc method, the optical bandgap energy ($E_{g}$) of ZnO nanostructured films could be calculated [53]. Using the recorded absorbance (A) spectra and film thickness, the absorption coefficient (*α*$=2.303\frac{A}{d}$) was estimated and used for the calculation of the direct optical band gap energy ($E_{g}$) [53]:

(1) $$\alpha hv=\sqrt{B}{\left(hv-E_{g}\right)}^{1/2}$$

where *hυ* is the incident photon energy.

The energy intercept of a plot of (*αhv*)^2^ vs. *hv* yields *E_g_* values for nanoparticulate and nanorod-arrayed films as shown in Figure 3c,d. The optical band gap energies for ZnO nanoparticulate and ZnO nanorod-arrayed films are 3.272 ± 0.003 and 2.992 ± 0.007 eV, respectively. This decrease may be related to the decrease in the oxygen vacancies as indicated by the EDX analysis. According to the PL spectra in Figure 2a, the nanorod-arrayed film had a lower density of oxygen vacancies than the nanoparticulate film, which was evidenced by a decrease in the intensity of the wide emission peak at around 570 nm [44]. As a result, the ZnO nanorod-arrayed film is better suited for solar energy applications such as photoelectrochemical (PEC) hydrogen production.

### 3.2. PEC H_2_ Production

The PEC current density–voltage (J_ph_-V) and current density–time (J_ph_-t) behaviors were tested using an OrigaFlex electrochemical station (OGFEIS attached to an OGF500 Pack). These behaviors were recorded using a cell with two electrodes. A 1 cm^2^ ZnO film was used as the working electrode, with a Pt electrode serving as the auxiliary electrode. As a solvent, these electrodes were submerged in 0.5 M Na_2_SO_3_.7H_2_O solution. Linear Sweep Voltammetry (LSV) readings were taken in the dark and under white light illumination of 100 mWcm^2^ from 0 to 1 V with a sweep rate of 10 mV/s. LSV observations were performed using the light source provided with a set of UV and visible optical filters from 390 to 636 nm. The amperometry mode was used to measure the J_ph_-t curves at 1 V for 900 s.

#### 3.2.1. PEC Current Density–Voltage (J_ph_-V) Behaviors

**Figure 4:**
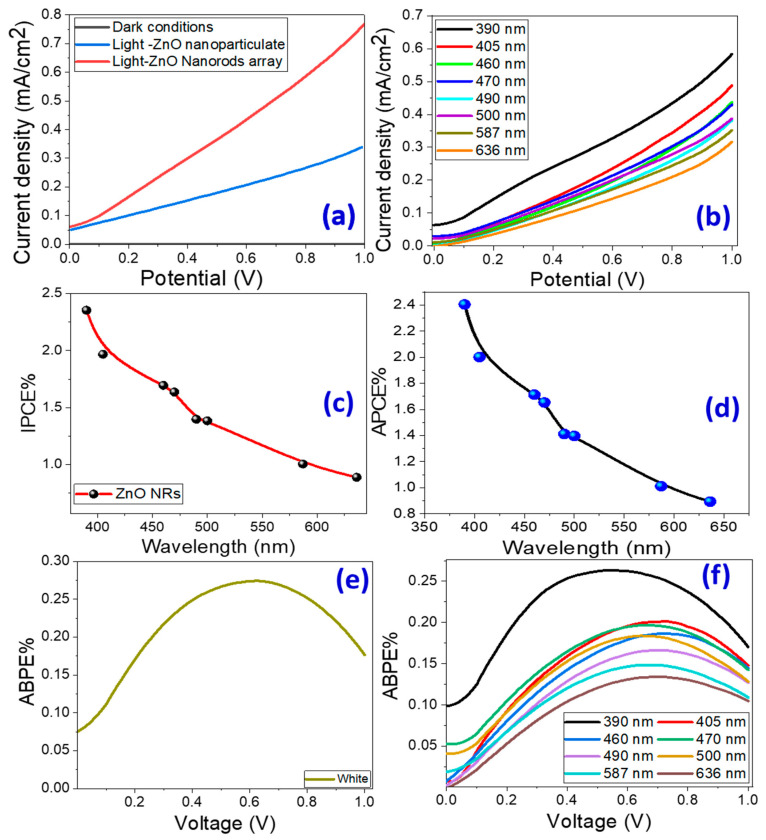
Current density versus applied voltage under (**a**) dark and white light illumination for nanoparticulate and nanorod-arrayed photoelectrodes, and (**b**) monochromatic light illumination. Conversion efficiencies of PEC process: (**c**) IPCE% and (**d**) APCE% versus the incident monochromatic wavelength. (**e**,**f**) ABPE% versus the applied voltage under white light and monochromatic light using the nanorod-arrayed film.

Figure 4a shows the PEC J_ph_ − V characteristics of ZnO nanoparticulate and ZnO nanorod-arrayed films in dark and under white light illumination. The J_ph_-V curve indicates that as the positive potential rises, J_ph_ values climb rapidly, indicating that the electrode was constructed of an n-type semiconductor with electrons as the preponderance of free carriers. As seen, the ZnO nanorod-arrayed film has a high photocurrent density, which reaches 0.77 mA/cm^2^ at 1 V versus 0.35 mA/cm^2^ for nanoparticulate morphology and 6.4 μA/cm^2^ in dark conditions. This result indicates an increase in the negative charge carriers in ZnO, implying that ZnO nanorods are more capable of producing hydrogen. The low value of the dark current density indicates that few chemical reactions occurred in the dark, and the high chemical stability of the produced ZnO nanorod-arrayed film in the Na_2_SO_3_·7H_2_O electrolyte versus the nanoparticulate ZnO photoelectrode, as shown by the reusability study in the Supplementary Materials (Figure S2). This implies that there is insufficient formation of electron–hole pairs and a well-formed depletion layer in the dark. Based on the difference in current densities in dark and white light, the ZnO nanorod-arrayed film is a more effective and durable photoelectrode than the nanoparticulate film. Figure S3 (Supplementary Materials) illustrates the photocatalytic mechanism for hydrogen production via water splitting utilizing ZnO nanorod-arrayed electrode. When ZnO is illuminated with photons of energy larger than its band gap, the electrons (e^−^) of the valance band (VB) are moved to the conduction band (CB), leaving an equivalent number of holes (h+) in the VB [ZnO +hυ → ZnO + e^−^ + h^+^]. The electrons migrate to the metal surface where they react with the adsorbed hydrogen ions to produce hydrogen gas [2H^+^ + 2e^−^ → H_2_]. Whereas, electron donors, such as SO_3_^2−^, react with the holes remaining in ZnO’s valence bands to form a variety of sulfur oxide ions [54]. As a recent alternative approach for overall water splitting from a kinetics perspective, a two-step/two-electron process has been investigated and has shown exciting progress [55]. In this process, water undergoes a redox reaction to produce hydrogen and hydrogen peroxide, the latter of which then decomposes to produce water and oxygen [2H_2_O + 2e^–^/h^+^ → H_2_ + H_2_O_2_; H_2_O_2_ → H_2_O + 0.5O_2_].

#### 3.2.2. Effect of Monochromatic Light Illumination and Conversion Efficiencies

Figure 4b shows J_ph_ versus the applied voltage under the monochromatic light illumination for the nanorod-arrayed ZnO photoelectrode in a 0.5 M Na_2_SO_3_·7H_2_O solution at RT. Bandpass filters with wavelengths ranging from 390 to 636 nm were used. Figure 4b shows that the highest photocurrent was obtained at 390 nm with J_ph_ = 0.583 mA/cm^2^. At 636 nm, the lowest current is observed, with J_ph_ = 0.316 mA/cm^2^. This J_ph_-λ dependence is related to the absorption behavior of the ZnO photoelectrode at each wavelength and supports the photoelectrode’s PEC catalytic response for the H_2_ generation process. In general, this behavior confirms the photoelectrode’s response and its ability to absorb a large portion of the solar spectrum in the UV/visible region.

The conversion efficiency of incident photons to current (IPCE%) by photoelectrodes at different wavelengths was estimated to be 1 V vs. RHE. This measurement helps determine how effectively photons are converted to current [56]. The IPCE% is calculated at 1 V using Equation (2) [57,58]:

(2) $$\mathrm{IPCE}\%=1240\cdot \frac{J_{\mathrm{ph}}}{\lambda \cdot \rho }\cdot 100$$

where ρ is the lamp’s illuminating light power density as a function of wavelength (see Supplementary Materials, Table S1). Figure 4c shows how IPCE% varies with wavelength. The highest IPCE% for the nanorod-arrayed ZnO photoelectrode is 2.35% at 390 nm, which correlates to the photoelectrode’s maximum absorption; it then decreases to 1.69% at 460 nm.

In the IPCE calculations, optical loss effects such as incident photon transmission or reflection were ignored. To correct optical losses, the internal quantum efficiency, also known as the absorbed photon-to-current conversion efficiency (APCE), must be calculated. APCE% is defined as the number of generated carriers that contribute to the generated photocurrent/absorbed photon. It is calculated using Equation (3) [56]:

(3) $$\mathrm{APCE}(\lambda )=\frac{\mathrm{IPCE}\left(\lambda \right)}{A\left(\lambda \right)}$$

where A(λ) is the optical absorption. Figure 4d demonstrates the change of APCE% as a function of the incident λ. The highest APCE% values for the ZnO nanorod-arrayed electrode were 2.4% at 390 nm and 2.0% at 405 nm, respectively.

To completely assess the PEC hydrogen generation of the ZnO nanorod-arrayed electrode, we evaluated the applied bias photon-to-current efficiency (ABPE%) as a function of applied voltage. The observed ABPE% was calculated using Equation (4) [59]:

(4) $$\mathrm{ABPE}\%=1240\cdot \frac{J_{\mathrm{ph}}}{\rho }\cdot \left(1.23-V_{\mathrm{app}}\right)\cdot \;100$$

where 1.23 denotes the standard state reversible potential of H_2_O and |Vapp| denotes the applied voltage during J_ph_ measurement. Figure 4e,f depicts the ABPE% versus applied voltage under white and monochrome light irradiation with the nanorod-arrayed electrode. The ABPE% rose as the applied voltage grew until it reached its highest value at the optimized voltage and then declined. Figure 4e shows that the greatest ABPE%(0.274) was obtained at 0.626 V under white light irradiation. Under monochromatic illumination, the maximum ABPE% (0.134) is obtained at 0.679 V for 636 nm and 0.263 at 0.555 V for 390 nm. As the incident wavelength decreased, the maximum ABPE% shifted to a lower applied voltage.

#### 3.2.3. PEC Stability of the Photocathode and Tafel Slope

As shown in Figure S2 (Supplementary Materials), the reusability study of the nanoparticulate ZnO and ZnO nanorod-arrayed photoelectrodes for 10 runs under white light illumination at 1 V indicates the high stability of the ZnO nanorod-arrayed photoelectrode. After 10 runs, the nanorod-arrayed photoelectrode still has 96.6% of its initial J_ph_, compared to 87.4% for nanoparticulate ZnO photoelectrode.

**Figure 5:**
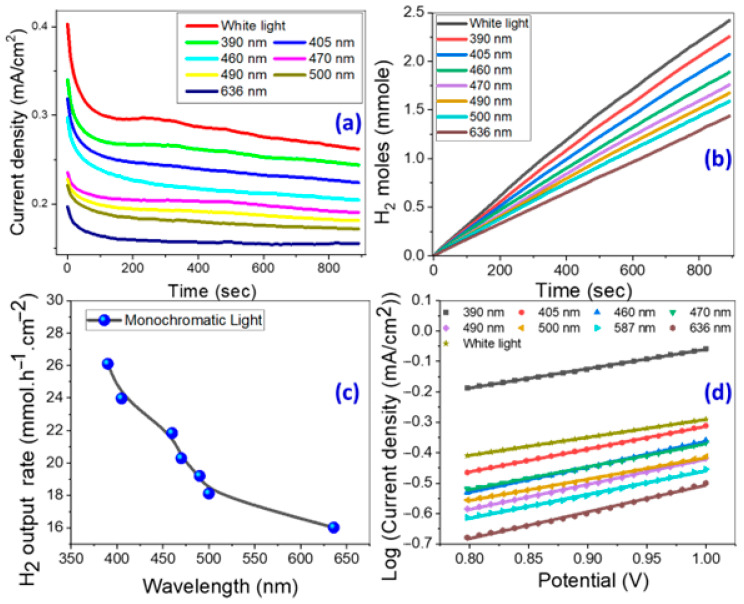
(**a**) Current density and (**b**) produced H_2_ moles versus PEC reaction time under white light and monochromatic light illumination, (**c**) H_2_ output rate versus the monochromatic wavelength, and (**d**) Tafel plot of log (current density) versus the applied potential using the nanorod-arrayed film.

The rise in photostability of the designed electrode over time, as shown in Figure 5a and Figure S4 (Supplementary Materials), is another significant element in increasing the efficacy of the PEC hydrogen generation system. Because of the low photo-erosion process that occurred during the first period, the J_ph_ value decreases very rapidly to achieve a steady value [60,61]. Due to higher ionic charge accumulation, the J_ph_ values indicate a very steady photoresponse of approximately 0.263 mA/cm^2^ at 900 s under white light irradiation versus 0.244 mA/cm^2^ under 390 nm illumination and 0.225 mA/cm^2^ under 405 nm illumination. After 600 min of illumination with white light and 390 nm monochromatic light, Figure S4 shows nearly steady photocurrent densities of 0.247 and 0.242 mA/cm^2^, respectively, indicating the great stability of the proposed electrode. A high photogenerated current density in light is a good predictor of high-performance PEC hydrogen generation [6,62]. It could be because the E_g_ value in the nanorod-arrayed electrode approaches 2.99 eV, which accelerates the redox reaction rate and thus encourages the PEC reaction, in addition to increasing the tunneling activity of the photogenerated carriers. The stability of the electrode structure was confirmed by illustrating the XRD patterns of the nanorod-arrayed ZnO electrode before and after PEC hydrogen production in Figure S5 (Supplementary Materials). These XRD patterns indicated that there was no obvious change to the ZnO nanorod-arrayed electrode as a result of the PEC application.

The quantity of produced hydrogen moles from the PEC process was theoretically determined using Faraday’s law, Equation (5) [63];

(5) $$H_{2}(\mathrm{moles})=\int_{0}^{t}\frac{J_{\mathrm{ph}}\mathrm{dt}}{F}$$

where F = 9.65 × 10^4^ C/mol and t is the reaction time. Figure 5 depicts the amount of produced H_2_ moles as a function of time based on the observed J_ph_-time data in Figure 5a,b. Figure 5c shows the expected hydrogen generation rate versus incident monochromatic light wavelength. For white light and 390 nm monochromatic illumination, the output rates were 28.43 and 26.11 mmol h^−1^cm^−2^, respectively. For comparison, Table 2 compares our photocatalyst’s obtained number of H_2_ moles and generated J_ph_ to other benchmarked semiconductor and ZnO nanostructured catalysts in the literature for hydrogen generation [64,65,66,67,68,69,70,71,72,73,74,75,76,77,78,79,80,81]. The nanorod-arrayed photoelectrode showed greater current density and hydrogen moles in this study than the published PEC catalysts in Table 2 [64,65,66,67,68,69,70,71,72,73,74,75,76,77,78,79,80,81]. According to the findings, the nanostructured ZnO nanorod-arrayed photoelectrode has better light-harvesting abilities, a high hydrogen production rate, and enhanced nanomorphological and nanostructural characteristics that are extremely advantageous for practical uses.

Light absorption efficiency is one of the main variables adding to the overall solar-to-hydrogen conversion efficiency (STH) for a solar energy-driven hydrogen production device [82]. The benchmark efficiency, or STH (solar to hydrogen efficiency), is the percentage of total H_2_ energy produced to total sunshine energy (AM 1.5 G, 0.1 W/cm^2^). Equation (6) can be used to determine STH% [83]:

STH = [(H_2_/S × (2.37 × 1.5 J/mol)]/[A × *P*] (6)

where *P* and A are the light power density in mW/cm^2^ and the illuminated area of the photoelectrode in cm^2^, respectively. H_2_/S is the production rate of the hydrogen moles in mmol/sec. The STH values for the ZnO nanorod-arrayed electrode were 3.72% and 3.12% under 390 and 405 nm monochromatic illumination, respectively.

The anodic Tafel slope (β) is estimated from the Tafel plot, Figure 5d, to define the HER mechanism and the rate-limiting period, according to the following relationship: V = β log(J_ph_) + c [84]. Perfect PEC catalysts have excellent current exchange rates and a low β, which add to high HER efficacy [85]. At various monochromatic wavelengths, electrode-specific data such as corrosion current (I_corr_), anodic Tafel slope (β), and correlation coefficient (R^2^) are calculated and displayed in Table 3 [86,87]. The slopes on the Tafel can be used to understand the reaction processes and rate-limiting stages in the PEC process. For example, if recombination is the limiting phase and the Tafel slope is 30 mV/decade, the Volmer–Tafel process will prevail. The Volmer–Heyrovsky hydrogen evolution process is likely to prevail when PEC desorption is the limiting step and the Tafel slope is 40 mV/decade. If the Tafel slope is 120 mV/decade, the reaction pathways are determined by the surface infused with adsorbed hydrogen. The nanorod-arrayed photoelectrode had low corrosion currents, as shown in Table 3. The corrosion rate is proportional to I_corr_. As a result, low I_corr_ indicates a low corrosion rate and, therefore, high durability of the proposed electrode. In summary, the suggested nanorod-arrayed photoelectrode is an efficient and stable photoelectrode for PEC H_2_ production when compared to previously reported ZnO nanostructures, Table 2. This is due to its wide and low bandgap energy, improved structural properties such as the low density of dislocations and preferred growth along the (002) plane, the low Tafel slope, and low corrosion rate.

## 4. Conclusions

In this study, nanoparticulate and nanorod-arrayed films have been designed to better understand how nanomorphology can impact structural, optical, and PEC hydrogen production efficiency, as well as electrode stability. The ZnO nanorod-arrayed film was produced using the CBD method from 0.05 M zinc acetate precursor over a period of 2 h. Spray pyrolysis at 300 °C for 15 min with an 80 µL/min discharge rate was used to generate the nanoparticulate film. Various characterization methods were used to investigate the morphologies, structures, elemental analysis, and optical characteristics. The XRD showed the growth of a preferred c-axis oriented wurtzite ZnO nanorod-arrayed film in the (002) orientation plane and nanoparticulate along (101) orientation. While the crystallite size for the (002) orientation of the wurtzite hexagonal nanorods was 100.8 nm, it was 42.1 nm for the (101) nanoparticulate ZnO. For orientation (101) of nanoparticulate and (002) of nanorods, the minimum dislocation values are 5.6 × 10^−^^4^ and 1.0 × 10^−^^4^ dis/nm^2^, respectively. The band gap is decreased from 3.27 eV to 2.99 eV by changing the surface morphology from nanoparticulate to hexagonal nanorod array. The PEC generation of H_2_ using the proposed photoelectrodes was investigated under white and monochromatic light irradiation. Under 390 and 405 nm monochromatic light, the solar-to-hydrogen conversion efficiency for the ZnO nanorod-arrayed electrode was 3.72% and 3.12%, respectively. For white light and 390 nm monochromatic illuminations, the output H_2_ production rates were 28.43 and 26.11 mmol·h^−1^·cm^−2^, respectively. After 10 reusability cycles, the nanorod-arrayed photoelectrode maintains 96.6% of its initial photocurrent, compared to 87.4% for the nanoparticulate ZnO photoelectrode. The lowest Tafel slope (58.71 mV/dec) was observed under white light irradiation, compared to 63.7 and 87.91 mV/dec under 390 nm and 636 nm monochromatic light illumination, respectively. The computed conversion efficiencies, output H_2_ generation rates, Tafel slope, and the corrosion current demonstrate how the nanorod-arrayed morphology provides excellent PEC performance and durability. These findings are particularly significant because they offer a low-cost design strategy that may be considered to produce efficient photoelectrodes with acceptable stability for green PEC hydrogen generation. To create more efficient and stable photoelectrodes for our upcoming research, we intend to use a plasmonic nanolayer or a doping strategy, which can increase the commercial viability of the designed photoelectrodes.

## Figures and Tables

**Table 1:** The XRD structural parameters of ZnO nanoparticulate and nanorod-arrayed films.

Sample	2θ°	(hkl)	Rel. Intensity	DC (nm)	Dislocation Density (dis/nm^2^) × 10^−4^	TC	Lattice Parameters
ZnO Nanoparticulate	31.8	(100)	75.0	47.5	4.4	1.54	a = 2.436 Å c = 5.212 Å V = 26.79 Å^3^ Zn-O bond length = 1.683 Å in-plane stress = −4.69 × 10^8^ N/m^2^
	34.4	(002)	77.9	75.9	1.7	1.60	
	36.2	(101)	100.0	42.1	5.6	2.05	
	47.7	(102)	20.2	36.8	7.4	0.41	
	56.6	(110)	26.0	40.8	6.0	0.53	
	62.9	(103)	19.2	21.9	20.9	0.39	
	68.0	(112)	23.1	94.9	1.1	0.47	
ZnO Nanorods	31.8	(100)	47.9	56.5	3.1	0.88	a = 2.436 Å c = 5.198 Å V = 26.72 Å^3^ Zn-O bond length = 1.680 Å in-plane stress = 7.97 × 10^8^ N/m^2^
	34.5	(002)	100.0	100.8	1.0	1.83	
	36.3	(101)	49.7	51.9	3.7	0.91	
	47.6	(102)	46.4	39.2	6.5	0.85	
	56.7	(110)	45.8	59.1	2.9	0.84	
	62.9	(103)	47.3	81.2	1.5	0.86	
	68.0	(112)	45.8	32.0	9.7	0.84	

**Table 2:** Values of photocurrent density and the number of hydrogen moles of our ZnO nanorod-arrayed photoelectrode compared with other benchmarked photocatalysts and ZnO nanostructured electrodes reported in the literature for H_2_ production.

Nanostructure	Electrolyte	Photocurrent Density	No. of H_2_ Moles	Ref
ATO/PANI/PbS	Sewage water	0.13 mA/cm^2^ @1 V	0.1 mmol/cm^2^ h	[64]
SnO_2_-ZnO QuantumDots/g-C_3_N_4_ (SZ/g-C_3_N_4_)	5% aqueous glycerol solution	-	13,673.61 μmol g^−1^/5 h	[65]
PMT/roll-GO	Sewage water	0.098 mA/cm^2^ @1 V	10 μmole/h·cm^−2^	[66]
SnO_2_	Na_2_SO_4_	0.1 mA/cm^2^ @1 V	-	[67]
GE/Calix/Cyn/IrO_2_	BS (pH 7.4) containing 10 mM NaCl and 0.1 mM MgCl_2_	0.182 mA/cm^2^ @1 V	1.25 × 10^−8^ mol cm^−2^	[68]
BiVO_4_	0.5 M KPi + 0.5 M Na_2_SO_3_	0.55 mA/cm^2^ @1 V	-	[69]
CuWO_4_	pH10 buffer solution	0.5 mA/cm^2^ @1 V	-	[70]
ZnO Film	0.5 M NaClO_4_	0.045 mA/cm^2^ @1 V	-	[71]
ZnO Nanorods	0.5 M Na_2_SO_4_	0.089 mA/cm^2^ @0 V		[72]
ZnO Nanowires	0.1 M Na_2_SO_4_	0.05 mA/cm^2^ @0 V		[73]
ZnO Nanorods	0.5 M Na_2_SO_4_	0.25 mA/cm^2^ @0.25 V SCE		[74]
ZnO Nanotubes	0.1 M KH_2_PO_4_	0.36 mA/cm^2^ @0.5 V SCE		[75]
ZnO Nanotubes	0.1 NaOH	0.312 mA/cm^2^ @0 V SCE		[76]
Fe_3_O_4_-ZnO Core–shell	0.1 KOH	0.52 mA/cm^2^ @1.23 V		[77]
ZnO Triangles	1 M NaOH	0.321 mA/cm^2^ @1.23 V		[78]
ZnO Nanoparticles	0.1 M NaOH	0.105 mA/cm^2^ mA/cm^2^ @0 V SCE		[79]
ZnO Nanowires/ RGO/ZnIn_2_S_4_	1.0 M Na_2_SO_4_	0.6 mA/cm^2^ @0.5 V		[80]
ZnO Nano pencil arrays	0.5 M Na_2_SO_4_	0.7 mA/cm^2^ @1 V		[81]
ZnO Nanorod-arrayed electrode	0.5 M Na_2_SO_3_·7H_2_O	0.77 mA/cm^2^ @1 V	28.43 mmol h^−1^cm^−2^	This work

**Table 3:** Values of corrosion current and Tafel slope at different monochromatic wavelengths.

Sample	I_Corr_(μA·cm^−2^)	β (mV·dec^−1^)	R^2^
390 nm	201	63.70 ± 0.39	0.99928
460 nm	88	83.43 ± 0.61	0.99957
405 nm	63	74.33 ± 0.36	0.99898
470 nm	76	75.09 ± 0.84	0.99761
490 nm	58	82.55 ± 0.81	0.99817
500 nm	76	70.22 ± 0.82	0.99739
587 nm	58	77.98 ± 1.05	0.99659
636 nm	41	87.91 ± 1.31	0.99581
White light	132	58.71 ± 0.10	0.99996

## Data Availability

Not applicable.

## Supplementary Materials

The following supporting information can be downloaded at https://www.mdpi.com/article/10.3390/mi14051047/s1, Figure S1. Histogram for pore diameter distribution, whereas the pore diameter is ranged from 10 nm to 28 nm with an average value of 19 nm. Figure S2: The stability study of photocurrent density as a function of the reusability of (a) nanoparticulate ZnO photoelectrode and (b) ZnO nanorods arrayed photoelectrode for 10 runs under white light illumination at 1V. Figure S3. Schematic diagram of the photocatalytic hydrogen production utilizing ZnO nanorod-arrayed film. Figure S4. Full-length current density versus PEC reaction time under white light and monochromatic light illumination using the nanorod-arrayed film. Figure S5. XRD patterns of ZnO nanorod-arrayed film before and after PEC application. The XRD pattern of the ZnO nanorod arrayed film after PEC H2 generation indicated that there was no obvious change to the ZnO electrode as a result of the PEC application. Table S1: Light power intensity of Xenon lamp at different monochromatic wavelengths.
