# Supplementary material for: Photoelectrochemical Green Hydrogen Production Utilizing ZnO Nanostructured Photoelectrodes
Source: Micromachines (Basel). 2023 May 14;14(5):1047. doi: 10.3390/mi14051047 (PMC10224315; doi:10.3390/mi14051047)
Supplement: Supplementary file 1 [file micromachines-14-01047-s001.zip › micromachines-2345937-supplementary.pdf]

Photoelectrochemical green hydrogen production utilizing ZnO nanostructured photoelectrodes

Sameerah I. Al-Saeedi

Department of Chemistry. collage of Science, Princess Nourah bint Abdulrahman University, P.O.Box 84428, Riyadh 11671, Saudi Arabia

E-mail: sialsaeedi@pnu.edu.sa

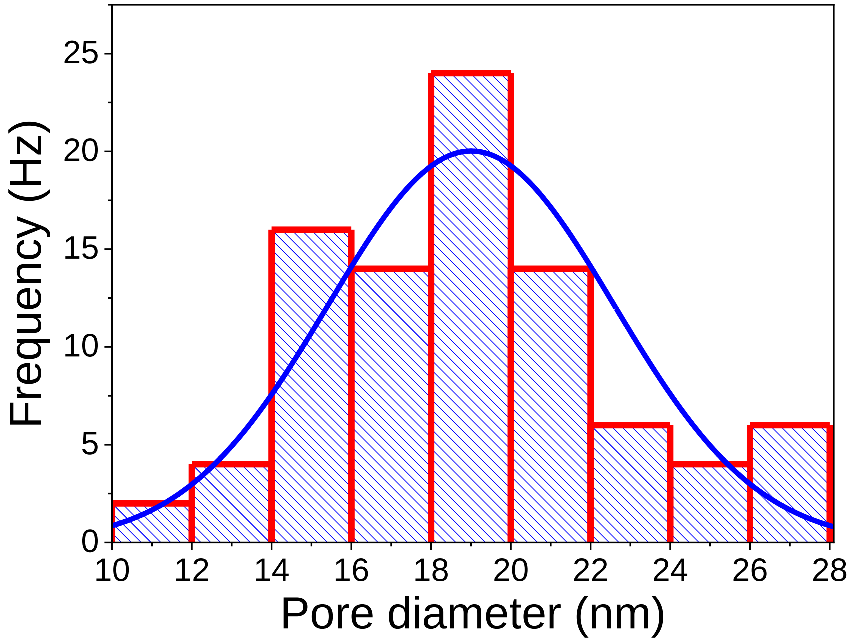

Figure S1. Histogram for pore diameter distribution, whereas the pore diameter is ranged from 10 nm to 28 nm with an average value of 19 nm.

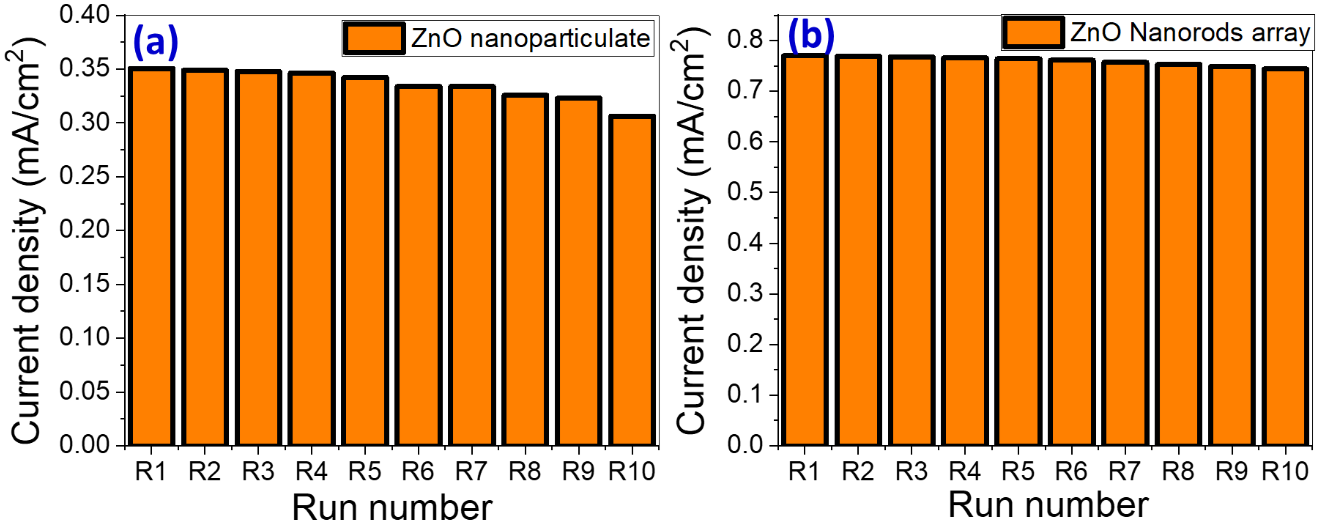

Figure S2: The stability study of photocurrent density as a function of the reusability of (a) nanoparticulate ZnO photoelectrode and (b) ZnO nanorods arrayed photoelectrode for 10 runs under white light illumination at 1V.

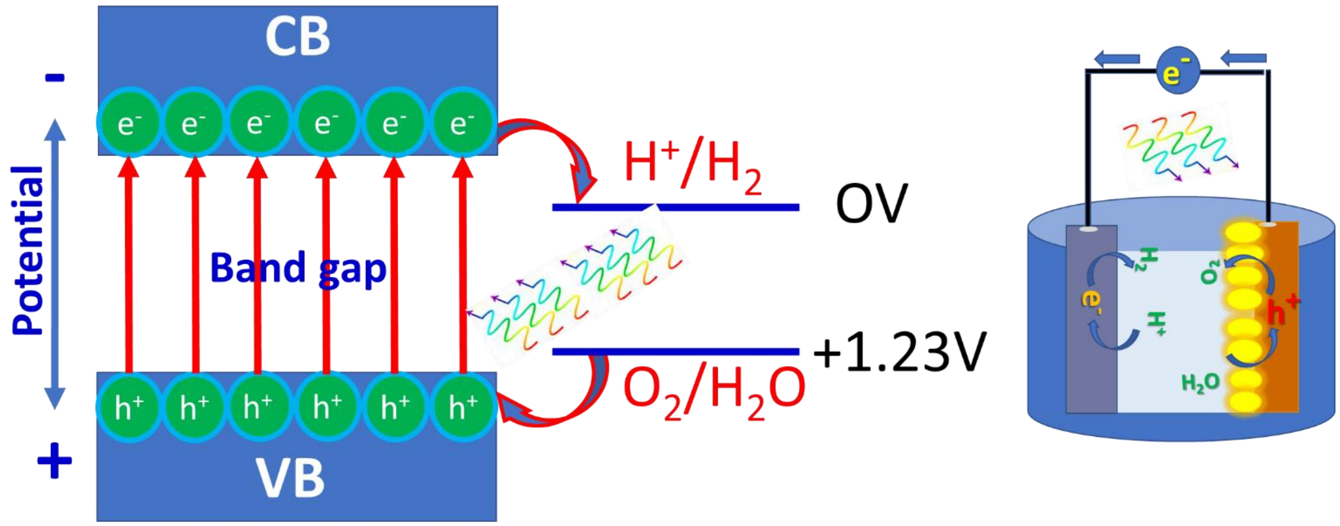

Figure S3. Schematic diagram of the photocatalytic hydrogen production utilizing ZnO nanorod-arrayed film,

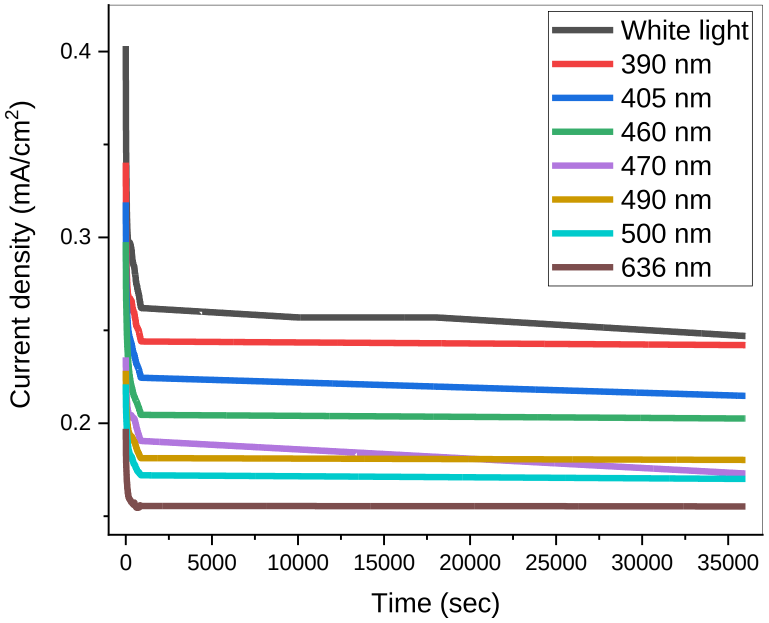

Figure S4. Full-length current density versus PEC reaction time under white light and monochromatic light illumination using the nanorod-arrayed film.

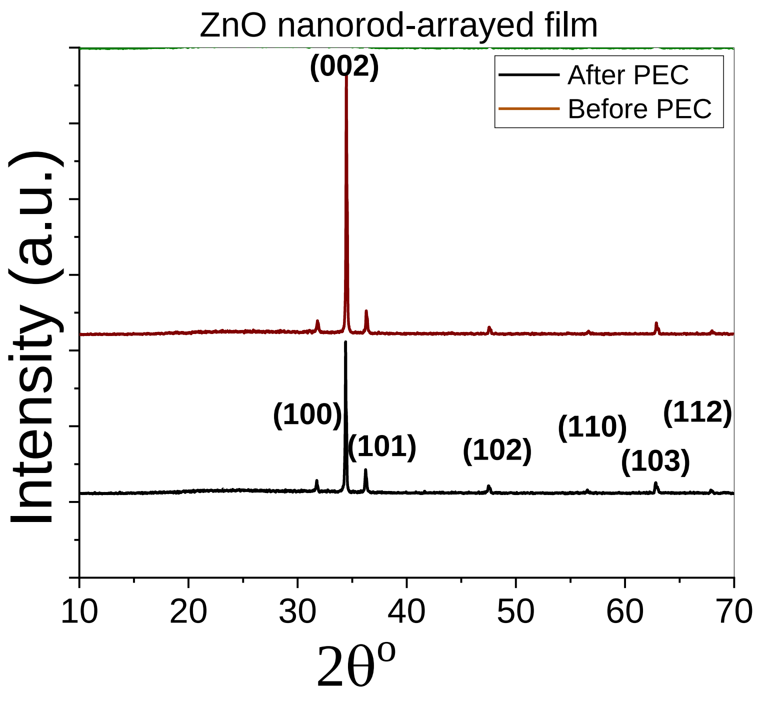

Figure S5. XRD patterns of ZnO nanorod-arrayed film before and after PEC application. The XRD pattern of the ZnO nanorod arrayed film after PEC H_2 generation indicated that there was no obvious change to the ZnO electrode as a result of the PEC application.

Table S1: Light power intensity of Xenon lamp at different monochromatic wavelengths:

Wavelength(nm)	P(mW/cm^2)
390	78.75
405	75.96
460	69.36
470	69.36
490	69.36
500	69.36
508	69.108
588	74.01
636	69.36
